# Supplementary material for: Causal Relationship between Aging and Anorexia Nervosa: A White-Matter-Microstructure-Mediated Mendelian Randomization Analysis
Source: Biomedicines. 2024 Aug 16;12(8):1874. doi: 10.3390/biomedicines12081874 (PMC11351342; doi:10.3390/biomedicines12081874)
Supplement: Supplementary file 1 [file biomedicines-12-01874-s001.zip › Supplementary Figure/Figure S8/Leaveoneout plot of MR analysis for TL to ubm-b-1982.pdf]

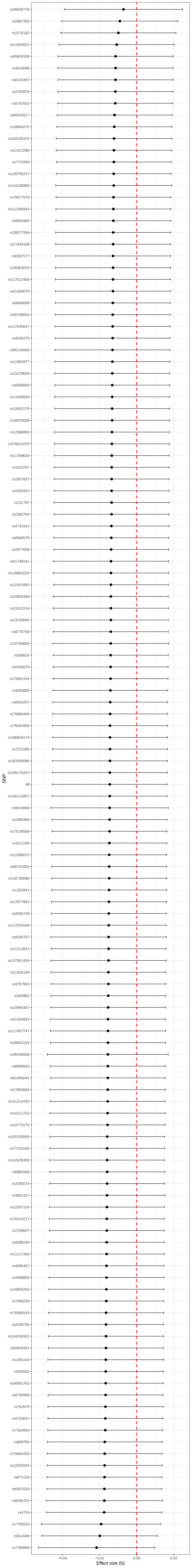

rs35640778
rs2967355
rs2276182
rs11584821
rs45604339
rs4616688
rs8102497
rs2763979
rs5742915
rs80324517
rs10840270
rs202034370
rs11412296
rs7772289
rs139795227
rs143190905
rs79977579
rs112394943
rs9955360
rs28577594
rs17445108
rs6587577
rs28363070
rs117512405
rs41269079
rs9940099
rs56799554
rs117630647
rs4530278
rs80116508
rs11991877
rs11579626
rs6659669
rs11699829
rs12932179
rs16978028
rs12369950
rs376641875
rs11769630
rs1023767
rs1957937
rs1003322
rs131797
rs2282764
rs4731541
rs6584579
rs2977608
rs61748181
rs116863223
rs12451892
rs10805346
rs12412214
rs13230646
rs6776756
rs10768683
rs939916
rs2293579
rs73581419
rs3093888
rs6054257
rs76666449
rs78491606
rs188918174
rs7221585
rs182059586
rs185174247
All
rs145114957
rs9419958
rs1985369
rs73730598
rs1611236
rs11085072
rs66731853
rs142730696
rs1332941
rs17677991
rs2056726
rs117034449
rs8105767
rs11212631
rs137901416
rs11426156
rs3767952
rs450962
rs10845387
rs41304832
rs117407747
rs28502153
rs35446936
rs6669563
rs61405042
rs17803849
rs141214782
rs10112752
rs10773176
rs150150565
rs77231040
rs142426306
rs6881568
rs3785074
rs3891167
rs11557154
rs76219171
rs7209057
rs9398196
rs11117354
rs4695407
rs4498805
rs10905255
rs7099229
rs76065543
rs2538745
rs144204502
rs59409453
rs1291143
rs932002
rs56061761
rs6790988
rs762679
rs4743037
rs7164950
rs869785
rs75664430
rs12925933
rs871134
rs6007020
rs6536702
rs4724
rs7705526
rs611646
rs7790856
SNP
−0.04
−0.02
0.00
0.02
Effect size (b)
